# Bacterial acetate metabolism and its influence on human epithelia

**DOI:** 10.1042/ETLS20220092

**Published:** 2023-03-22

**Authors:** Jennifer Hosmer, Alastair G. McEwan, Ulrike Kappler

**Affiliations:** School of Chemistry and Molecular Biosciences, Australian Infectious Diseases Research Centre, The University of Queensland, St. Lucia, Australia

**Keywords:** acetate, host–microbe interactions, metabolism, microbiology, short-chain fatty acid

## Abstract

Short-chain fatty acids are known modulators of host–microbe interactions and can affect human health, inflammation, and outcomes of microbial infections. Acetate is the most abundant but least well-studied of these modulators, with most studies focusing on propionate and butyrate, which are considered to be more potent. In this mini-review, we summarize current knowledge of acetate as an important anti-inflammatory modulator of interactions between hosts and microorganisms. This includes a summary of the pathways by which acetate is metabolized by bacteria and human cells, the functions of acetate in bacterial cells, and the impact that microbially derived acetate has on human immune function.

## Introduction

Short-chain fatty acids (SCFAs) are molecules with fewer than six carbon atoms in the aliphatic tail, and in biological systems the most commonly found representatives are acetate (C2), propionate (C3), and butyrate (C4). These SCFAs are ubiquitously found in natural environments, produced by bacterial, fungal, and mammalian cells during anaerobic fermentation and aerobic fermentative respiration [1–10].

In the human body, SCFAs are found in the highest concentrations in the intestinal tract, where bacteria metabolize indigestible saccharides and other molecules and release SCFAs as end products. SCFA concentrations in the intestine range from 20 to 140 mM, with acetate accounting for 60–75% of this and exceeding propionate and butyrate at least twofold in concentration [11,12]. It has been estimated that 36% of colonic-derived acetate becomes systemically available, reaching 50–200 μM in venous serum [11,13–18]. In other bacterially colonized body sites such as the oral cavity and urogenital tract, acetate is detected at 6–38 mM and up to 120 mM (depending on the presence or absence of infection/inflammation), respectively [19]. Acetate is particularly suited to exerting systemic effects, as, unlike propionate or butyrate, it can traverse cell membranes without requiring a specific uptake system [20,21].

## Bacterial acetate metabolism pathways are redundant and tightly regulated

**Figure 1.**
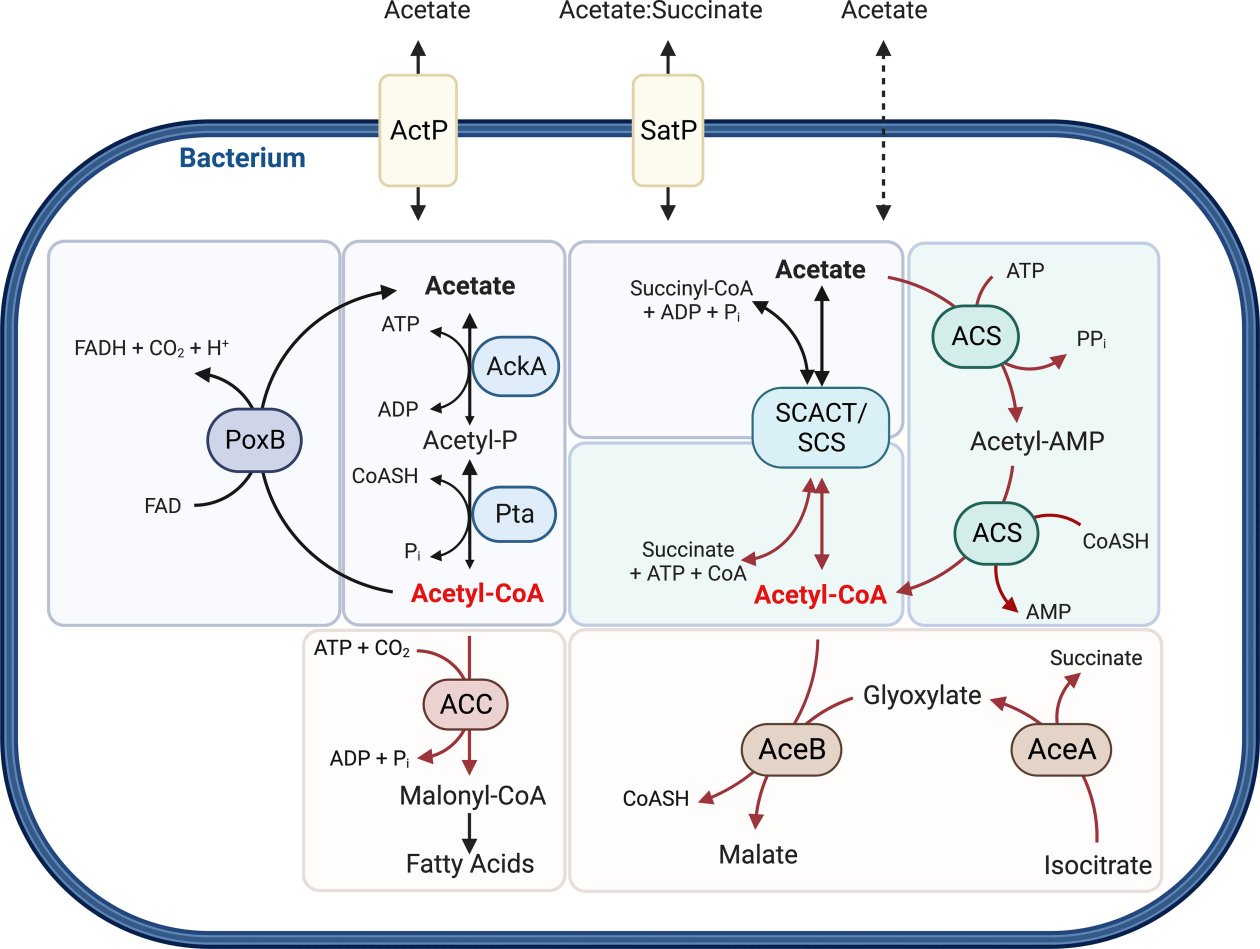
Schematic representation of the bacterial acetate metabolism pathways, at which acetyl-CoA (red) is a central metabolite. Acetate can be imported into the cell by acetate permease (ActP), an acetate/succinate symporter (SatP), and/or passive diffusion. *Acetate formation* (blue) can occur via the reversible acetate kinase (AckA) and phosphotransacetylase (Pta) reaction, a pyruvate:menaquinone oxidoreductase (PoxB), or the reversible succinyl-CoA : Ac-CoA transferase and succinyl-CoA synthetase (SCACT/SCS). *Acetate assimilation into Acetyl-CoA* (green) can occur via acetyl-CoA synthetase (ACS) and/or SCACT/SCS. *Assimilation of Acetyl-CoA into biomass* (yellow) can use multiple pathways, including assimilation into fatty acids (ACC, acetyal-CoA carboxylase) of TCA cycle intermediates (AceA, isocitrate lyase; AceB, malate synthase). Abbreviations: AMP, adenosine monophosphate; ADP, adenosine diphosphate; ATP, adenosine triphosphate; CoASH/CoA, coenzyme A; P_i_, phosphate; PP_i_, diphosphate; FAD, flavin adenine dinucleotide (quinone); FADH, flavin adenine dinucleotide (semiquinone); CO_2_, carbon dioxide; H^+^, proton. This figure was created using BioRender.

Acetate production by bacteria is mediated by two main pathways that often occur together in the same microorganism and show functional redundancy. These pathways either involve acetate kinase and phosphotransacetylase (AckA-Pta), a set of reactions that allows ATP production via substrate-level phosphorylation, or a pyruvate : menaquinone oxidoreductase denoted PoxB or CidC [22–35] (Figure 1). In addition to passive membrane permeability, acetate release and uptake can be effected by either an acetate permease (encoded by *actP*) or an acetate/succinate symporter (encoded by *satP*) [36,37] (Figure 1).

While acetate is mostly a metabolic endproduct in bacteria, there are also several mechanisms by which bacteria can assimilate acetate into biomass, usually via the formation of acetyl-coenzyme A (Ac-CoA) that can then be assimilated via the glyoxylate cycle and the TCA cycle, contributing to biosynthetic intermediate production and energy generation [32,33,38–41]. A typical enzyme in Ac-CoA production is Ac-CoA synthetase (ACS, also known as Ac-CoA ligase), which is found in diverse bacteria such as *Enterobacteria*, *Pseudomonads*, *Neisseria* spp., and *Mycobacterium* spp. [24–32,34,39–49] (Figure 1). An additional, reversible pathway that uses succinyl-CoA : Ac-CoA transferase and a succinyl-CoA synthetase has been identified in flagellate protozoans [50], eukaryotes [51,52], and bacteria [53,54] (Figure 1). In bacteria, the succinyl-CoA : Ac-CoA transferase can be used to consume acetate, e.g. in *Acetobacter* [54] while *Cutibacteria* spp., present on skin and mucosal surfaces, use it to produce acetate [53]. Other mechanisms for acetate assimilation include the methyl malonyl-CoA pathway [55–57] or glutamate-dependent acetate uptake into the TCA cycle in *Neisseria meningitidis*, an organism that lacks isocitrate lyase and malate synthase [32,34] (Figure 1). The AckA-Pta pathway that usually leads to acetate production has also been shown to be reversible. However, it exhibits low overall activity in the reverse direction [42–45].

For the majority of bacteria, acetate is not the most preferred carbon source, and its utilization can be subject to catabolite repression if glucose is present [26,27,58–62]. There is also evidence of RpoS-mediated activation of acetate consumption during post-exponential growth [59,63–67]. During anaerobic/fermentative growth, up-regulation of *ackA-pta* expression has been linked to the global anaerobic regulator Anr (Fnr homolog) and integration host factor subunit alpha (lhfA) in bacteria such as *Pseudomonas* spp. and *Neisseria* spp. [31,34,66,68], but there are also other known regulators, such as the CrbS/R two-component system [28,29] that controls acetate consumption via ACS, and a LysR-type transcriptional regulator, CidR, for acetate production via the pyruvate:menaquinone oxidoreductase [22,69,70].

While many bacteria can tolerate acetate reasonably well, acetate accumulation can impair growth and inhibit the production of proteins and plasmid DNA [71–76]. This is best studied in *Escherichia coli* and common human pathogens, *Staphylococcus* spp. and *Pseudomonas* spp., where acetate metabolism results in intracellular acidification and respiratory inhibition [22,23,59,60,66,70,76–78]. Additionally, the production of Ac-CoA and acetyl-phosphate can alter protein acetylation non-enzymatically, which has been shown to modulate bacterial virulence and metabolism [79,80].

## Acetate is a host cell nutrient involved in epithelial barrier integrity

As in bacteria, acetate is also a host cell nutrient, and its role has been best studied in the tumor microenvironment [81]. Tumor cells show increased glucose uptake and pyruvate formation due to the predominance of ‘Warburg’ metabolism. This results in a metabolic imbalance with excess carbon being directed to lactate and acetate formation via pyruvate decarboxylases or hydrolysis reactions that use protein deacetylases and Ac-CoA hydrolase [4,81].

External acetate, that can be present both in a tumor environment and as a result of microbial action, is transported into mammalian cells by members of the monocarboxylate transporter family, where it can then be converted to Ac-CoA by Ac-CoA synthetases (ACSS) that are present in both mitochondrial (ACSS1) and cytosolic forms (ACSS2) [4,81]. The acetate-derived Ac-CoA can be used for ATP production, protein acetylation via lysine acetyltransferases, and fatty acid synthesis [4,82,83].

In addition to being an energy source, acetate has also been shown to have beneficial effects on epithelial integrity [84,85]. In intestinal epithelial cells, acetate triggers NLRP3 inflammasome activation, which, as shown in *nlrp*3^−/−^ mice, plays a key role in protection against colitis [86]. Wound healing, tight junction repair, and changes to the actin cytoskeleton can also be induced by acetate [87–90].

## Acetate is detected by GPCR-dependent and independent mechanisms

Most molecular effects of acetate are mediated by G-protein coupled receptors (GPCRs), expressed on intestinal enterocytes and other cell types found throughout the body, that can sense environmental acetate [91–95]. Both GPCR43 (also known as free fatty acid receptor (FFA2)) and GPCR41 (also known as FFA3) can sense acetate, but most acetate signaling is mediated by GPCR43 that, unlike GPCR41, binds acetate preferentially over other SCFAs. GPCR-dependent signaling mediates acetate-based chemoattraction [12,92,96,97] but has also been shown to suppress chemoattractant ligands (CCLs) CCL1 and CCL2 and cytokine-induced neutrophil chemoattractants (CINC) CINC-1 and CINC-2αβ [88,92,98–101]. Additional mechanisms for sensing acetate are likely to exist, as GPCR43-independent acetate signaling via an as-yet-unknown mechanism has been demonstrated [102].

## Acetate affects host cell inflammation through post-translational modification of histones and the NLRP3 inflammasome

**Figure 2.**
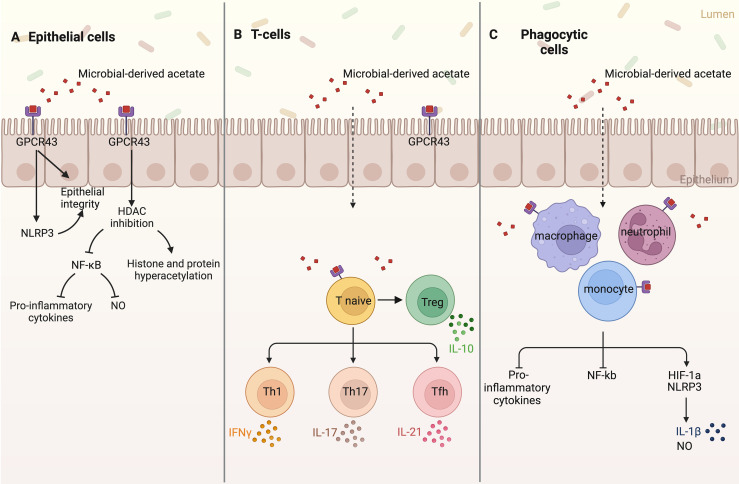
Schematic representation of the G-protein coupled receptor (GPCR) 43-dependent and independent acetate signaling pathways in host epithelia and immune cells. (**A**) Acetate signaling in host epithelial cells triggers NLRP3 and histone deacetylase (HDAC) inhibition, resulting in increased epithelial integrity and wound healing, reduced expression of pro-inflammatory cytokines and nitric oxide (NO), and histone and non-histone protein hyperacetylation. (**B**) Acetate triggers the differentiation of naïve T-cells to T-regulatory (Treg) and T-helper (Th1, Th17, and Tfh) cells, increasing the expression of interferon-gamma (IFNγ), interleukin 17 (IL-17), interleukin 21 (IL-21), and interleukin 10 (IL-10). (**C**) Phagocytic cells including macrophages, neutrophils, and monocytes, after sensing acetate, reduce the expression of pro-inflammatory cytokines and NF-κβ but increase the expression of NLRP3 and HIF-1α, resulting in increased interleukin 1β (IL-1β) and NO production. This figure was created using BioRender.

High concentrations of acetate in or around the host cell can result in changes in protein acetylation following Ac-CoA formation via ACSS1/ACSS2 [103–105]. Acetylation is a common post-translational modification on a wide range of histone and non-histone proteins [106–108]. Acetylation of histones changes gene expression [106], while acetylation of non-histone proteins affects their subcellular localization, DNA binding, transcriptional activity, protein–protein interaction, and stability [107,108]. Protein acetylation depends on the formation of Ac-CoA from acetate and is moderated by histone acetylases (HACs) and histone deacetylases (HDACs) which are ubiquitously expressed by host cells [18,109,110]. Previously it had been believed that acetate has no HDAC modulatory activity. However, there is increasing evidence that histone hyperacetylation, particularly on histones H3 and H4, may not only be due to increased Ac-CoA concentrations, but is promoted by acetate-mediated inhibition of HDACs [18,109–115]. HDAC inhibition has been linked to NF-κB inactivation and the subsequent suppression of pro-inflammatory cytokines, including TNF-α, IL-1β, IL-18, and IL-6, and nitric oxide (NO) production in different human epithelial cells (Figure 2) [18,88,99,116].

This acetate-based suppression of pro-inflammatory cytokines, primarily TNFα, IFN-γ, IL-1α, IL-6, IL-12, and IL-18, and NF-κβ activation via HDAC inhibition has been demonstrated in activated macrophages, monocytes, and neutrophils (Figure 2) [18,86,98,102,117–121]. In contrast, HIF-1α and the NLRP3 inflammasome are activated by acetate, particularly lower concentrations (0.2–2 mM), as observed in neutrophils [122], leading to increased IL-1β and NO production (Figure 2) [102,123]. However, NLRP3 expression can be down-regulated by acetate signaling under hypoxic conditions, demonstrating that environmental oxygen availability influences downstream responses to acetate, and this should be investigated further [99,100].

The presence of acetate also increases the production of prostaglandin E [2] (PGE(2)), which can have both pro- and anti-inflammatory effects in neutrophils, monocytes, and T cells [98,124]. Lastly, while acetate appears to have no effect on dendritic cell development and function [125], it has been shown to modulate the function of CD4^+^ and CD8^+^ T cells through mTOR activation [110,126,127], PGE(2) up-regulation, and HDAC inhibition, specifically increasing the expression of the anti-inflammatory IL-10 [110,124]. Additionally, acetate aids CD4^+^ T cell differentiation to Th1 and Th17 effector cells, which results in increased expression of IFN-γ and IL-17, respectively (Figure 2) [110,124].

## Acetate modulates inflammation in major microbially colonized organs

Being primarily a microbial-derived metabolite, immunomodulatory effects of acetate generally occur in the host where there is extensive microbial colonization, including major organs such as the skin, oral cavity, gastrointestinal (GI), urogenital and respiratory tracts [128–132]. Acetate-mediated host–microbe interactions are essential for the functioning of several physiological host processes, including tissue development, nutrient absorption and metabolism, and the proper function of the immune system [12]. This has been best studied for the GI tract [87–90].

The nutrient-rich human GI tract is inhabited by complex bacterial communities reaching up to 10^11 ^bacteria/ml in the colon. In healthy individuals, these communities are dominated by *Firmicutes* and *Bacteroidetes* with lower abundances of *Actinobacteria* and *Proteobacteria* [128]. Small populations of organisms such as *Clostridioides difficile* that cause disease if they overgrow may also be present. The predominant acetate-producing bacteria are *Bacteroidetes* as well as *Prevotella* spp., *Bifidobacterium* spp., and *Akkermansia muciniphila* [1]. Acetate produced by these microbes can be used by GI tract inhabiting *Firmicutes* to produce butyrate, another SCFA [91,92]. The composition of gut bacterial communities can be impacted by host diet, where a high-fibre diet is associated with communities capable of greater SCFA production and other microbial-derived metabolites [133]. In addition to dietary effects, drug treatments such as the administration of antibiotics and host genetic factors can affect the prevalence of acetate-producing microorganisms in the GI tract [86,134].

In healthy hosts, the same immunomodulatory mechanisms that control inflammation in response to the normal microbiota also protect the bowel from invasion by pathogens [135]. In the GI tract, microbial-derived acetate is associated with epithelial maintenance, wound healing, and improved barrier function [87–90]. Acetate also lowers colonic inflammation in mice [19,71,86,136] and has been shown to exert a probiotic effect against enteropathogens [137]. Alterations in the microbial composition, particularly an increase in *Proteobacteria* and/or *Firmicutes,* have been linked to multiple pathologies, such as obesity, colorectal cancer, and inflammatory bowel diseases (IBD). In fact, acetate supplementation has been shown to alleviate the severity of IBD in acetate-fed, germ-free, *GPCR43^−/−^* mice and high-fibre diet mouse models [110,133,138].

The healthy skin microbiota have essential roles in protection against pathogens and the breakdown of natural products such as lipids and proteins [139]. Similar to the GI tract, skin microbiota are comprised of bacteria from the same four main phyla, Actinobacteria (36–51%), Firmicutes (24–34%), Proteobacteria (11–16%), and Bacteroidetes (6–9%) [132]. However, in contrast with the GI tract, Bacteroidetes are not dominant in a healthy skin microbiome. The specific composition of the microbiota differs depending on the physiology of the skin site, where, for example, humid skin sites primarily harbor *Staphylococcus* (*Firmicutes*) and *Corynebacterium* (*Actinobacteria*), while in oily sites, *Cutibacterium* (*Actinobacteria*) species are the most common [129,139]. In this environment, microbial-derived acetate, primarily from Cutibacterium acnes and *Staphylococcus epidermidis*, can decrease microbial biofilm formation; however, if present in excess, it can drive inflammation observed e.g. in acne [140,141].

In the respiratory tract, the microbiota composition and relative abundance of each microbe are isolation site-specific and reflect the health status and age of the host [142]. However, there are clear differences between the microbiota of the upper and lower respiratory tract (LRT). The upper respiratory tract (URT), which consists of the nasal and oral cavities, harbors commensals and opportunistic pathogens such as *Staphylococcus* spp., *C. acnes*, *Corynebacterium* spp., *Moraxella* spp., *Haemophilus* spp., and *Dolosigranulum* spp. [130,131]. In contrast, in the LRT, *Prevotella*, *Veillonella* spp., and *Streptococcus* spp. are the main bacterial colonizers in healthy individuals [131,143–146]. Opportunistic nasal pathogens such as *Streptococcus pneumoniae*, *Haemophilus influenzae*, and *Moraxella catarrhalis* are less commonly isolated from a healthy LRT, but their presence is associated with diseases such as cystic fibrosis, pneumonia, and chronic obstructive pulmonary disease [142,144–146].

*In vitro*, acetate can increase the ability of alveolar macrophages to kill bacteria and viruses, such as S*treptococcus* spp., *Staphylococcus* spp., *Klebsiella pneumoniae*, and respiratory syncytial virus (RSV) [102,117–119,147–149], i.e. it appeared to show pro-inflammatory rather than anti-inflammatory action in these assays. There are few detailed studies of the effects of acetate on lung microbiota; however, it is known that some common respiratory tract colonizers, such as *H. influenzae*, produce high amounts of acetate as a metabolic endproduct, while other species, such as *Moraxella*, use acetate as a preferred carbon source [150–154]. Additionally, increasing acetate concentrations in the LRT have been associated with dysbiosis and proposed to be drivers of persistent neutrophilic inflammation [146]. However, there is also evidence that the cellular effects of acetate can be actively modified by bacteria such as *H. influenzae*. A recent study showed that acetate (7 mM) had an anti-inflammatory effect on human bronchial cells when live *H. influenzae* were present, but increased inflammation when the bronchial cells were stimulated antigenically with heat-killed *H. influenzae* [150]. This suggests that active metabolic interactions between bacteria and epithelial cells may be required for an anti-inflammatory effect [150]. Similar results have also been reported for A549 lung carcinoma cells, where *H. influenzae* culture supernatants induced a pro-inflammatory response [151]. Given that *H. influenzae* is a commensal of the human nasopharynx, but not the LRT, one could speculate that in the nasopharynx *H. influenzae* acetate production is a common process that promotes persistence. In contrast, acetate is an uncommon metabolite in the LRT, and its production by *H. influenzae* or other microbes then drives dysbiosis and neutrophilic airway inflammation.

Compared with the well-documented role of microbial-derived acetate in the GI tract, the role of acetate and acetate-producing microbiota in the respiratory tract is less defined, with the switch between pro- and anti-inflammatory effects and the dependency on the type of bacterial species present requiring further examination.

Contrary to what has been observed for the human GI tract, skin, and respiratory system, acetate is not associated with a healthy microbiome in the human urogenital tract. Here, lactate is normally present in high concentrations and is produced by a combination of *Firmicutes*, *Lactobacillus* and *Streptococcus* spp., which are the dominant genera of the urogenital tract microbiota [132]. In addition to lactate, small amounts of acetate and succinate have also been observed, and their concentrations increase during disease [155]. During disease, such as neurogenic bladder dysfunction, interstitial cystitis, and urinary tract infection, there is a shift in the microbiota composition, with a particular increase in SCFA-producing bacteria, including *Lactobacillus, E. coli*, *K. pneumoniae*, *Proteus mirabilis*, *Enterococcus faecalis*, and *Staphylococcus saprophyticus* [156,157]. While little is known about the molecular effects of acetate on the epithelia of the urogenital tract, its presence is a consistent disease marker, and the measurement of acetate in vaginal fluid samples has even been suggested as a diagnostic tool for bacterial vaginosis [155].

In summary, while in the GI tract and skin acetate prevents inflammation and promotes the health of the host tissue, the role of microbial-derived acetate in the respiratory tract is ambiguous, and in the urogenital tract, it is a marker for inflammation. Further work is needed, especially in the respiratory and urogenital tract to clarify the association of acetate with disease development.

## Microbiota-derived acetate can have systemic effects on the human body

In addition to local effects on individual organ systems, metabolites such as acetate can drive cross-talk between GI tract microbiota and other organs, including the frequently discussed ‘gut-brain axis’ where acetate guides microglial maturation and regulation during disease [111,141,148,158–163]. In other organs, for example, despite evidence that local acetate production in the lung can drive dysbiosis [146], attenuation of allergic airway inflammation was observed in parallel with an increase in the gut of SCFA-producing *Bacteroidaceae* and *Bifidobacteriaceae*, particularly *Bifidobacterium longum* 5^1A^, enhanced by a high-fibre diet [164,165]. Similarly, a reduction in lung tissue injury during bronchopulmonary dysplasia was associated with an increase in gut *Ruminococcaceae*, known to be important SCFA producers [100]. It has been proposed, based on the studies presented above as well as extensive reviews [160,166–171], that this cross-talk is mediated by SCFAs that originate from the GI tract and circulate systemically, as well as the conditioning of GI tract immune cells by a healthy gut microbiota, resulting in the improved function of the overall immune system.

## Conclusions and research gaps

Despite being less studied than the more potent SCFAs, propionate and butyrate, recent studies have documented the importance of acetate homeostasis for maintaining a healthy state, particularly in the GI tract. This is mediated by bidirectional interactions between microbiota, host epithelia, and the host immune system, where, in many contexts, microbial-derived acetate reduced inflammation, improved epithelial barrier function, and increased wound healing capabilities [86–90]. Moreover, acetate, which acts primarily through GPCR43, in many cases, exerts an anti-inflammatory response by suppressing NF-κβ activation and HDAC activity to attenuate downstream production of pro-inflammatory cytokines in a wide variety of cell types and may be manipulated by host microbiota.

However, several aspects of these interactions need to be studied in more detail. Most research to date has been performed using *in vitro* or *ex vivo* infection models, often using only abiotic stimulation or treatment with exogenous acetate at different concentrations [41,70,102,116,121,149,172]. This makes understanding the systemic effects of acetate, why acetate in the respiratory tract and urogenital tract can be a disease marker, and the complex role of NLRP3 and how it responds to acetate challenging. To overcome this, the development of coculture models that use differentiated primary cells as well as using complex bacterial communities that reflect the *in vivo* environment and models that include epithelial and immune cells will be essential for future research in this field.

Additionally, while numerous studies explore the role of acetate in disease states for organ systems such as the URT/LRT or the urogenital tract, little is known about the role of acetate in healthy individuals. Particularly intriguing is the currently little-studied molecular basis for the ability of acetate to cause both pro- and anti-inflammatory effects. Similarly, more research is needed on the cross-feeding mechanisms between the gut microbiota and other host organs, and on the therapeutic potential of acetate in different disease models.

## Summary

- Acetate is an abundant, mostly microbial-derived SCFA in the human body.
- Acetate signaling occurs through GPCR43-dependent and independent methods and leads to suppression of HDAC and NF-κβ activity, thus exerting an anti-inflammatory effect.
- Despite the overall anti-inflammatory effects of acetate, conditions have been identified where acetate derived from human bacterial pathogens can trigger a pro-inflammatory response.
- Microbial-derived SCFAs from the GI tract can have systemic, immunomodulatory effects on other major host organs, including the respiratory tract.

## Open Access

Open access for this article was enabled by the participation of the University of Queensland in an all-inclusive *Read & Publish* agreement with Portland Press and the Biochemical Society under a transformative agreement with CAUL.

## Abbreviations

Ac-CoA: acetyl-coenzyme A
AckA-Pta: acetate kinase and phosphotransacetylase
ACS: Ac-CoA synthetase
CCL: chemoattractant ligand
CINC: cytokine-induced neutrophil chemoattractants
GI: gastrointestinal
GPCRs: G-protein coupled receptors
HDACs: histone deacetylases
IBD: inflammatory bowel diseases
LRT: lower respiratory tract
NO: nitric oxide
SCFAs: short-chain fatty acids
URT: upper respiratory tract
